# Psychometric Properties of the Chinese version of UPPS-P Impulsive Behavior Scale

**DOI:** 10.3389/fpsyt.2020.00185

**Published:** 2020-03-26

**Authors:** Yingying Zhang, Xian Qiu, Qihuan Ren, Zhirong Zhou, Huijing Zhou, Jiang Du, Valerie Voon, Chencheng Zhang, Wenjuan Liu

**Affiliations:** ^1^Department of Functional Neurosurgery, Ruijin Hospital, Shanghai Jiao Tong University School of Medicine, Shanghai, China; ^2^Department of Clinical Psychology, Shanghai Hongkou Mental Health Center, Shanghai, China; ^3^Department of Functional Clinic of the Community Based Methadone Maintenance Therapy in Shanghai Xuhui Mental Health Center, Shanghai, China; ^4^Department of Drug Dependence, Shanghai Yangpu Mental Health Center, Shanghai, China; ^5^Shanghai Mental Health Center, Shanghai Jiao Tong University School of Medicine, Shanghai, China; ^6^Department of Psychiatry and Behavioral and Clinical Neurosciences Institute, University of Cambridge, Cambridge, United Kingdom; ^7^Department of Psychological Medicine, Zhongshan Hospital, Fudan University, Shanghai, China

**Keywords:** UPPS-P Impulsive Behavior scale, impulsivity choice, gender difference, smoking status, China

## Abstract

**Objective:**

The present study aimed to assess the psychometric properties of a Chinese version of the UPPS-P Impulsive Behavior Scale. The associations between the UPPS-P scale and impulsivity choice, gender, smoking, and drinking status were also assessed.

**Methods:**

A total of 127 adults ranging from 21 to 65 years old participated in the study. Participants were administered with the Chinese version of the UPPS-P Impulsive Behavior Scale, Beck Depression Inventory II (BDI-II), and State–Trait Anxiety Inventory (STAI). Impulsivity choice tasks were also tested including the Delay Discounting Task (DDT), Balloon Analogue Risk Task (BART), and Beads Task (Beads).

**Results:**

A new version of the UPPS-P Impulsive Behavior Scale was formed that includes 40 items. The scores of the UPPS-P Impulsive Behavior Scale demonstrated adequate internal consistency on five subscales but less sufficient structure validity in the present sample. In addition, positive urgency was negatively related to the Beads task; negative urgency and positive urgency were positively related to the DDT and BART. Moreover, positive and negative urgency were positively correlated with depression; all five subscales were positively correlated with anxiety; sensation seeking was higher in males than females and in alcohol drinkers than non-drinkers; and lack of premeditation and lack of perseverance were higher in nonsmokers than smokers.

**Conclusions:**

The present study supports the reliability but not the structure validity of the UPPS-P Impulsive Behavior Scale. The impulsivity personality trait assessed by the UPPS-P scale was associated with impulsivity choice, depression, anxiety, gender, and drinking and smoking status. Further studies should be conducted to explore the structure of impulsivity in the Chinese population.

## Introduction

Impulsivity is a multifaceted construct that is generally characterized as a tendency to act prematurely without appropriate foresight or regard for potential consequences (1). It is very important in personality and plays a prominent role in many forms of dysfunctional behavior such as substance use disorders, eating disorders, and attention deficit hyperactivity disorder (2). Self-reported questionnaires including the UPPS-P Impulsive Behavior Scale and the Barratt Impulsivity Scale (BIS-11), or objective behavioral tasks measuring decisional and motoric forms of impulsivity, are used to assess impulsivity (3).

The UPPS-P Impulsive Behavior Scale consists of the UPPS Scale [Urgency, Premeditation (lack of), Perseverance (lack of), Sensation Seeking] and the Positive Urgency Measure. The UPPS scale is based on the Five Factor Model of personality and was developed by performing an exploratory factor analysis on some of the most common impulsivity measures, through which four distinct but associated impulsivity personality facets were finally formed (2). However, none of these four facets of impulsivity includes rash action under conditions of positive mood, which can produce increased risk behaviors. Therefore, Cyders et al. (4) developed a positive urgency measure to access the propensity to act rashly in response to positive affective state. The final UPPS-P scale can measure five facets of impulsivity, which are labeled negative urgency, lack of premeditation, lack of perseverance, sensation seeking, and positive urgency. Negative urgency refers to the tendency to act rashly under conditions of negative affective state. Lack of premeditation refers to the tendency to act without thinking and reflecting on the consequences. Lack of perseverance refers to the inability to remain focused on a task that may be boring or difficult. Sensation seeking refers to the tendency to seek out exciting activities or new experiences. Studies have shown that specific impulsivity traits are associated with specific behaviors (5, 6). For example, negative urgency was associated with aggression and lack of premeditation, and positive urgency was associated with smoking status, while sensation seeking and lack of perseverance were associated with problematic use of alcohol (7). There are also gender differences in impulsivity, such that studies show consistently higher levels of sensation seeking in males than females (8–14). However, there are mixed results for gender differences in the other four UPPS-P facets. Research on impulsivity and cigarette smoking has found that smokers are typically more impulsive than nonsmokers (5). Therefore, the UPPS-P scale is a useful tool for understanding and predicting different dysfunctional behaviors.

The UPPS-P scale is widely used for speakers of the English language and has been translated into several languages such as Italian and Spanish (15–17). The Chinese version of the UPPS scale and the short version of the UPPS-P scale have shown acceptable reliability and validity in college students (18, 19). However, the Chinese version of the UPPS-P scale has not been examined among Chinese adults with a wide range of ages. The aim of this study was to explore the psychometric properties of the scores of a Chinese version of the UPPS-P scale in a sample of adults. We performed item analysis and determined the reliability index to assess reliability. We then used confirmatory factor analysis to examine the validity of the five-factor model of the UPPS-P scale and explored the association between the UPSS-P scale and other measures of impulsivity to evaluate its construct validity. In addition, the differences in impulsivity between males and females, smokers and nonsmokers, and drinkers and nondrinkers were examined. We assumed that males would have higher scores on sensation seeking than females, smokers would have higher scores on lack of premeditation and positive urgency than nonsmokers, and drinkers would have higher scores on sensation seeking and lack of perseverance than nondrinkers. There was no particular hypothesis on the association between the UPPS-P scale and other measures of impulsivity because the results of previous studies are inconsistent.

## Methods

### Participants

The study was approved by the Ruijin Hospital Ethics Committee of Shanghai Jiao Tong University School of Medicine. Participants were enrolled at two sites, Ruijin Hospital, Shanghai Jiao Tong University School of Medicine, and Shanghai Hongkou Mental Health Center. Subjects meeting the following criteria were enrolled in the study: (1) more than 18 years old; (2) absence of psychological and mental disease: score on the Beck Depression Inventory II less than 29; (3) sufficient reading proficiency to complete questionnaires and computerized tasks; and (4) able to give written consent. Participants were recruited through advertisement by convenience sampling and included students, nurses, and technicians who study or work at the hospital or clinic, as well as family members, relatives, or friends of the experimenters. The participants were given 40 yuan as recompense.

A total of 127 participants completed the scales. There were two participants with BDI-II scores > 29. Therefore, 125 participants were included in the final analysis. Most participants were men (*n* = 79, 63.2%). The mean age was 46 years (standard deviation [SD] = 10.2; range, 21–65), and the mean duration of education was 11 years (SD = 4.2; range, 3–25). The majority of the sample was aged 30 to 60 (*n* = 109, 87.2%). The marital status of participants was as follows: single (*n* = 14, 11.2%), married (*n* = 105, 84.0%), and divorced (*n* = 6, 4.8%). The number of participants reporting smoking or drinking habits was 38 and 41, respectively.

### Measures

#### UPPS-P Impulsive Behavior Scale (UPPS-P)

The UPPS-P Impulsive Behavior Scale is a 59-item questionnaire assessing impulsive personality traits. Each item is rated on a four-point scale ranging from 1 (*agree strongly*) to 4 (*disagree strongly*) indicating the subject’s agreement with statements. This scale includes the following five subscales: negative urgency (NU), lack of premeditation (LPM), lack of perseverance (LPS), sensation seeking (SS), and positive urgency (PU) (2, 4). Each subscale has 10–14 items and a higher score represents a greater level of impulsivity. The items were translated from English into Chinese by a professional translator with a background in psychology, and then checked and revised by a senior psychologist fluent in both Chinese and English. Psychometric properties of the UPPS-P are reported in detail in the *Results* section.

#### Beck Depression Inventory II (BDI-II)

The Beck Depression Inventory II is a self-administrated 21-item scale detecting the presence of depression (20). The score of each item ranges from 0 to 3, with higher scores representing higher levels of depression severity. The internal consistency of BDI-II scores in this study was 0.838.

#### State-Trait Anxiety Inventory (STAI)

The State-Trait Anxiety Inventory consists of two 20-item self-reported subscales, which are STAI State (STAI-S) and STAI Trait (STAI-T) (21). STAI-S assesses a transient momentary emotional status and STAI-T targets the general reaction in stressful situations. Each item is rated on a four-point Likert scale, ranging from 1 (*not at all*) to 4 (*very much so*) for the STAI-S and 1 (*almost never*) to 4 (*almost always*) for STAI-T. The range of scores for each subscale is 20–80, where a higher score indicates greater anxiety. The internal consistency of the STAI total, STAI-S, and STAI-T scores were 0.913, 0.864, and 0.836, respectively.

#### Delay Discounting Task (DDT)

The Delay Discounting Task used in the study was computerized and consisted of 27 items measuring immediate gratification or the tendency to discount delayed rewards (22). Subjects were presented a hypothetical choice between a smaller, immediate monetary gain and a larger, delayed monetary gain. The task took about 3 min. Each question has a specific *k*-value, which ranges from 0 (selection of the delayed gain for all items) to 0.25 (selection of the immediate gain for all items), where a higher value of *k* corresponds to a higher level of impulsivity. The overall *k* value was used as a variable in the study.

#### Balloon Analogue Risk Task (BART)

The BART is a computerized measure of risk-taking behavior (23). In this study, the task was programmed and presented using software and consisted of 20 balloon trials. In each balloon trial, each pump will earn 5 cents and cause the balloon to inflate incrementally. The minimum and maximum pump of each balloon are 1 and 16, while the 16^th^ pump can cause the balloon to explode. If the participant chooses to cash out before the balloon exploding, then they collect the money earned for that trial, but if the balloon explodes, earnings for that trial are lost. Participants are not given any explicit information about the probability that the balloon will pop on a given trial. The BART took about 4 min. The average number of pumps adjusted for only unexploded balloons was used as the parameter of risk-taking, and higher scores indicate risker performance.

#### Beads Task (Beads)

The Beads Task used in the current study was a computerized measure of reflection impulsivity and consisted of two jars with an 80:20 blue to red vs. 80:20 red to blue ratio (24, 25). Participants were asked to pick beads one at a time from an unseen jar and instructed to use the sequence of picked beads to figure out or guess which jar the beads were likely picked from. The Beads Task took about 2 min. The measurement was the mean number of beads that participants used before making a decision. Higher scores represent less reflection impulsivity.

### Procedure

This study was approved by local institutional review boards, and all participants provided written informed consent before participating. All participants included in this study completed the self-report measures and most of them completed the DDT, BART, and Beads task. All the measures and tasks were administrated in a randomized order. Participants completed them individually in a quiet room. One experimenter instructed the participants on how to perform the behavioral task.

### Data analysis

The Kolmogorov–Smirnov one-sample test was used to test whether the scores of the different tests were normally distributed. Pearson correlation was used to examine the correlation between the scores of each item and intended subscales. We sorted the participants according to the total scores of each subscale such that the first 27% of the participants were assigned to the high group and the last 27% were assigned to the low group. An independent-sample *t*-test was used to test the difference between the two groups of subjects on the score of each item. We deleted items that were not distinguishable in the two groups or had an item-subscale correlation lower than 0.3. Confirmatory factor analysis (CFA) was conducted with maximum likelihood estimation to assess the five-factor structure of the new version of the UPPS-P. The maximum likelihood chi-square statistic, the Comparative Fit Index (CFI), and the root mean square error of approximation (RMSEA) were used to assess the goodness-of-fit of the model; CFI ≥.95 or RMSEA < .06 indicates good fit (26). We removed items with a standardized regression weight < .40. The internal consistency of the UPPS-P total and subscales was examined with Cronbach’s α, and the Pearson correlations between the UPPS-P total and subscale scores and other impulsivity measures were analyzed to explore construct validity.

To explore the relationship between demographic variables and UPPS-P, we used an independent-sample *t*-test to assess differences of the UPPS-P total and subscale scores between males and females, smokers and nonsmokers, and drinkers and nondrinkers. Statistical analyses were conducted using SPSS 18.0. A two-tailed value of *p* < .05 was considered statistically significant.

## Results

### CFA of the UPPS-P

**Figure 1:**
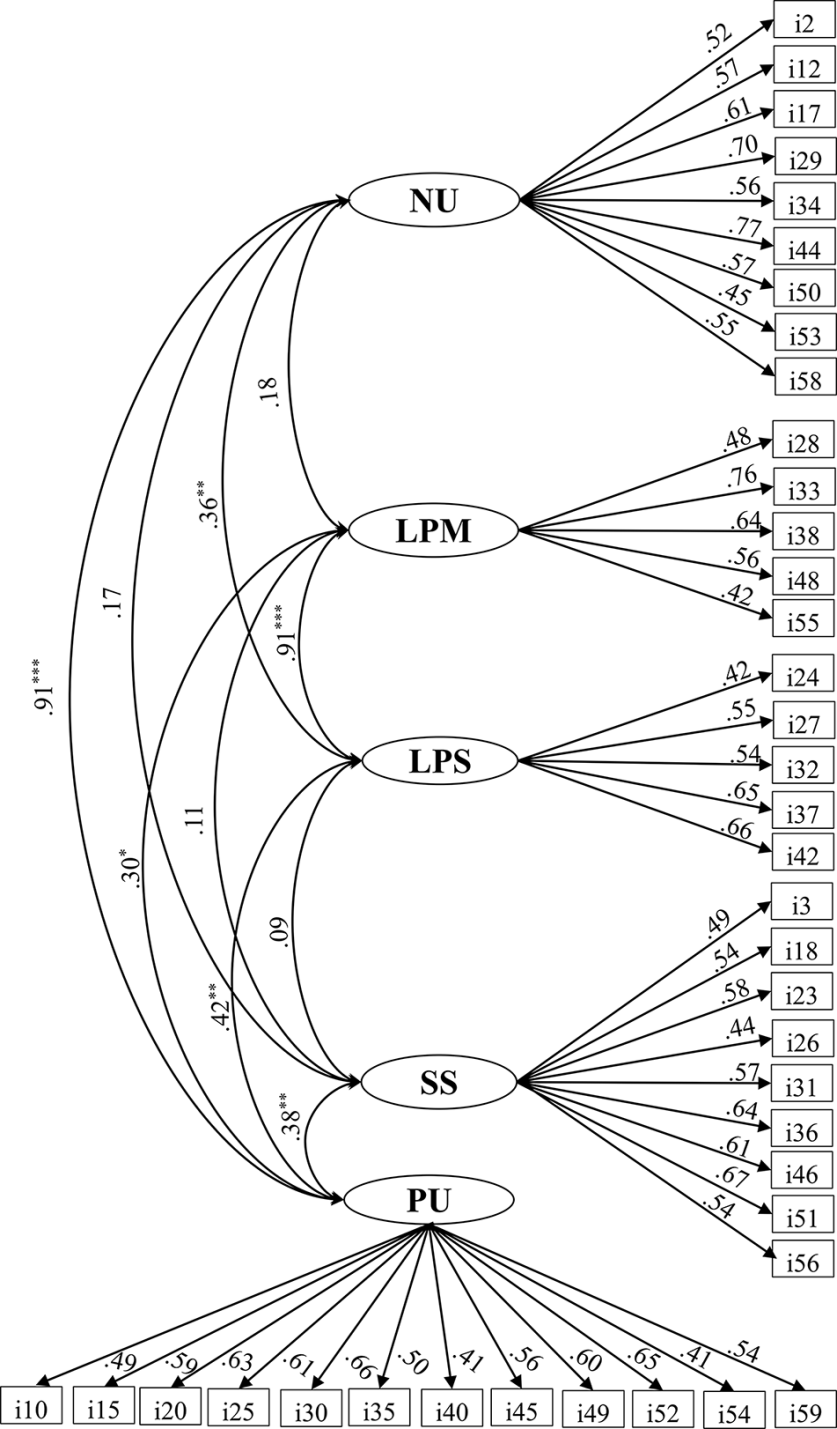
Five-factor model of the 40-item version of the UPPS-P. NU, Negative Urgency; LPM, Lack of Premeditation; LPS, Lack of Perseverance; SS, Sensation Seeking; PU, Positive Urgency.^*^p < 0.05, ^**^p < 0.01, ^***^p < 0.001.

The item-subscale correlation analysis demonstrated that Items 43 (*r* =.23) and 47 (*r* =.27) had low correlations with the intended subscale. Only Item 43 failed to distinguish between the high and low groups (*t* = 1.57, *p* > .05). We deleted Items 43 and 47 and conducted the CFA on the new version of UPPS-P. These CFA results indicated that the fit indices of the five-factor model were unacceptable for the UPPS-P (*χ*^2^ = 2521.87, *df* = 1529, *p* < .000, RMSEA =.072, CFI =.584). We deleted 15 items with a standardized regression weight < .40, which included Items 22 and 39 from the NU subscale, Items 1, 6, 11, and 16 from the LPM subscale, Items 4, 9, and 14 from the LPS subscale, Items 8, 13, and 41 from the SS subscale, and Items 5, 7, and 57 from the PU subscale. The standardized regression weights of the other items ranged from.40 to.78. The five-factor model of the remaining 42 items fitted the data better (*χ*^2^ = 1269.99, *df* = 809, *p* < .000, RMSEA =.068, CFI =.730). We also deleted Items 19 and 21 with standardized regression weights lower than.40. The standardized regression weights of the other 40 items ranged from.41 to.77. The five-factor model of the remaining 40 items (**Figure 1**) fitted the data better than the model with 42 items (*χ*^2^ = 1163.74, *df* = 730, *p* < .000, RMSEA =.069, CFI =.737). Thus, the NU, LPM, LPS, SS, and PU subscales included 9 (Items 2, 12, 17, 29, 34, 44, 50, 53, and 58), 5 (Items 28, 33, 38, 48, and 55), 5 (Items 24, 27, 32, 37, and 42), 9 (Items 3, 18, 23, 26, 31, 36, 46, 51, and 56), and 12 (Items 10, 15, 20, 25, 30, 35, 40, 45, 49, 52, 54, and 59) items, respectively.

### Reliability and correlations of the UPPS-P

A Kolmogorov–Smirnov test indicated that the scores of UPPS-P, NU, LPM, SS, PU, STAI-T, and BART were normally distributed, while the scores of LPS, BDI-II, STAI-S, DDT, and Beads showed nonnormal distributions. **Table 1** shows the means and standard deviations of the participants’ scores of all measures used in the study. The internal consistency of the UPPS-P was acceptable, with Cronbach’s α of.83,.70,.69,.81, and.84 for NU, LPM, LPS, SS, and PU, respectively. The scores of each item were significantly correlated with the scores of the UPSS-P and intended subscales.

**Table 1:** Pearson correlations and descriptive statistics for study variables.

	No. of Items	Range	M	SD	NU	LPM	LPS	SS	PU	UPPS-P
NU ^a^	9	9-33	17.3	5.62						
LPM ^a^	5	5-17	8.8	2.68	0.11					
LPS ^a^	5	5-17	8.5	2.68	0.23^**^	0.65^**^				
SS ^a^	9	9-33	18.1	5.98	0.15	0.06	0.03			
PU ^a^	12	12-39	19.7	6.25	0.73^***^	0.25^**^	0.33^***^	0.35^***^		
UPPS-P ^a^	40	43-121	72.4	15.8	0.76^***^	0.44^***^	0.51^***^	0.58^***^	0.88^***^	
DDT ^b^	/	0-0.25	0.05	0.08	0.22^*^	-0.03	0.07	0.13	0.26^**^	0.24^*^
BART ^c^	/	2.00-8.12	5.5	1.36	0.35^***^	0.02	0.15	-0.03	0.22^*^	0.23^*^
Beads ^d^	/	1-21	8.1	5.07	-0.13	0.02	-0.11	-0.18	-0.21^*^	-0.21^*^
BDI-II ^a^	21	0-26	7.5	6.52	0.39^***^	0.07	0.16	0.11	0.45^***^	0.39^***^
STAI-S ^a^	20	20-50	30.9	8.26	0.52^***^	0.28^***^	0.32^***^	0.20^*^	0.52^***^	0.57^***^
STAI-T ^a^	20	21-57	35.3	8.61	0.51^***^	0.27^**^	0.35^***^	0.22^*^	0.55^***^	0.59^***^
Age	/	/	/	/	-0.02	-0.21^*^	-0.09	-0.06	0.09	-0.04
Years of Education	/	/	/	/	-0.03	0.17	-0.08	0.04	-0.14	-0.04

M, Mean scores; SD, Standard Deviation; DDT, Delay Discounting Task; BART, Balloon Analogue Risk Task; Beads, Beads Task; BDI-II, Beck Depression Inventory II; STAI-S, State Subscale of State–Trait Anxiety Inventory; STAI-T, Trait Subscale of State–Trait Anxiety Inventory; NU, Negative Urgency; LPM, Lack of Premeditation; LPS, Lack of Perseverance; SS, Sensation Seeking; PU, Positive Urgency.

^a^n = 125, ^b^ n = 111, ^c^ n = 102, ^d^ n = 109.

*p < 0.05, ^**^p < 0.01, ^***^p < 0.001.

**Table 1** presents the correlations among the scores of the five subscales in terms of the internal construct of the UPPS-P. All five subscale scores were significantly correlated with the UPPS-P total scores (*r* =.44–.88, *p* < .001). In the current version of the UPPS-P, PU was strongly correlated with NU, moderately correlated with LPS and SS, and weakly correlated with LPM. LPS was strongly correlated with LPM and weakly correlated with NU, but not significantly correlated with SS. However, there were no significant correlations between SS and the other subscales except PU. In addition, LPM was not notably correlated with NU.

**Table 1** also shows the correlations between scores on the UPPS-P and other measures assessing depression (BDI-II), anxiety (STAI), and impulsivity (DDT, BART, and Beads). PU was moderately correlated with BDI-II, STAI, and DDT, weakly correlated with BART, and negatively correlated with Beads. NU was moderately correlated with BDI-II, STAI, and BART and weakly correlated with DDT, but was not significantly correlated with Beads. STAI was moderately correlated with LPM and LPS, and was weakly correlated with SS. LPM, LPS, and SS were not significantly correlated with BDI-II, DDT, BART, or Beads.

### Correlations between UPPS-P and demographic variables

The UPPS-P total score and all subscale scores were not significantly correlated with the years of education (**Table 1**). Only the scores of the LPS subscale were negatively correlated with age (*r* = −.21, *p* < .05). **Table 2** shows the means and standard deviations of the participants’ scores on the UPPS-P subscales by gender, drinking, and smoking status. The scores of the SS subscale differed significantly between males and females, and between drinkers and nondrinkers. The scores on the LPM and LPS subscales differed significantly between nonsmokers and smokers.

**Table 2:** Means and standard deviation of participants’ scores of the UPPS-P subscales on demographic variables.

Measures	Gender			Smoking Status			Drinking Status		
	Male	Female	p value	Nonsmokers	Smokers	p value	Nondrinkers	Drinkers	p value
NU	16.8 ± 5.32	18.1 ± 6.07	NS	17.4 ± 5.87	17.0 ± 5.05	NS	17.3 ± 6.02	17.3 ± 4.77	NS
LPM	8.5 ± 2.59	9.3 ± 2.77	NS	9.1 ± 2.73	8.1 ± 2.43	0.049	8.9 ± 2.73	8.6 ± 2.59	NS
LPS	8.2 ± 2.58	9.1 ± 2.78	NS	8.9 ± 2.73	7.7 ± 2.41	0.040	8.6 ± 2.63	8.3 ± 2.82	NS
SS	19.0 ± 5.86	16.6 ± 5.94	0.031	17.6 ± 6.09	19.3 ± 5.60	NS	17.3 ± 5.97	19.8 ± 5.70	0.029
PU	19.9 ± 6.22	19.4 ± 6.36	NS	19.6 ± 6.34	20.0 ± 6.11	NS	19.6 ± 6.62	20.0 ± 5.50	NS

NS, Not Significant; NU, Negative Urgency; LPM, Lack of Premeditation; LPS, Lack of Perseverance; SS, Sensation Seeking; PU, Positive Urgency.

## Discussion

This study aimed to assess the psychometric properties of the scores of a Chinese version of the UPPS-P Impulsive Behavior scale. We found that the CFA results were not sufficient to support the original structure of the UPPS-P scale. We deleted 19 items to obtain a 40-item scale. The five-factor model of the 40-item scale fitted the data better than did the 59-item scale. Overall, the 40-item scale demonstrated good psychometric properties. First, the five subscales and UPPS-P total scores showed good internal consistency. The item analyses also showed significant item-total and item-subscale correlations. Second, according to the correlations between different subscales, SS was not correlated with other subscales except PU, NU had the highest association with PU, and LPS had the highest association with LPM. Moreover, PU and the UPPS-P total scores had significant correlations with DDT, BART, and Beads; NU had a significant correlation with DDT and BART; and LPM, LPS, and SS had no significant correlations with DDT, BART, and Beads. Fourth, NU and PU were positively related to depression and all the subscales were positively related to anxiety. Regarding relationships between the UPPS-P scale and demographic variables, LPS was negatively correlated with age; SS was higher in males than females and drinkers than nondrinkers; and LPM and LPS were higher in nonsmokers than smokers.

The associations between sensation seeking and the other four subscales were similar to the associations in the short versions of the French and English translations of the UPPS-P (9, 27). In contrast to lack of premeditation, perseverance, and negative urgency, sensation seeking seemed to assess a specific facet of impulsivity that was unrelated to these three subscales. In addition, negative urgency was not notably correlated with a lack of premeditation; this has not been reported in previous studies. However, the low correlation between negative urgency and the lack of perseverance is common (27–29). The relationship between the various facets of impulsivity may vary in different countries because of cultural diversity.

We explored the relationship between the five subscales and other measures of impulsivity. The DDT, measuring decision-making impulsivity, was positively related to negative urgency and positive urgency. The BART, measuring risk-taking behaviors, was also positively related to negative urgency and positive urgency. Negative urgency and positive urgency were distinct factors loading onto a higher “emotion-based rash action” trait in previous studies (9, 27). Delay discounting and risk-taking were supposed to assess the similar aspects of impulsivity measured by the NU and PU subscales. The Beads task, measuring reflection impulsivity, was found to be negatively related to positive urgency. Different measures of impulsivity can be divided into three categories: impulsivity personality traits, choices, and actions, which showed moderate intercorrelations (30). Our study used three measures of impulsivity choice that had different correlations with the impulsivity personality trait. Therefore, further studies are needed to explore the relationships among impulsivity personality traits, choices, and actions so as to gain a better understanding of dysfunctional behaviors.

We also explored the relationship between the five subscales and the variables for depression, anxiety, and demography. The results of our study and earlier studies suggest that the impulsivity trait can be influenced by depression and anxiety (9, 14, 28). In addition, some studies showed that impulsivity decreased as age increased, which was found here for lack of perseverance (8, 31). Moreover, males scored significantly higher on sensation seeking than did females, which was in accordance with previous research findings (8, 10, 13, 14). However, some studies found that males scored higher than females on positive and negative urgency, but the results of other studies were the opposite (8, 10, 13, 14, 31). That our study gave different results may be because of the unbalanced sample in terms of gender. Furthermore, cigarette smokers had a lower level of impulsivity than nonsmokers, which is inconsistent with the findings of other studies in adolescents and adults (5, 32, 33). A meta-analysis of impulsivity-related traits and cigarette smoking in adults indicated that smoking status and severity of nicotine dependence were significantly associated with all impulsivity-related traits (5). Moreover, lack of premeditation and positive urgency showed the largest association with smoking status, while positive urgency showed the largest association with the severity of nicotine dependence. Besides this, alcohol drinkers had a higher level of sensation seeking than nondrinkers in our sample. However, some studies found that the other impulsivity was associated with drinking status, e.g., negative urgency and lack of premeditation were related to alcohol dependence (11, 27). The difference may be due to our small sample and lack of measures of smoking or drinking.

There are several limitations to our study. First, the sample was not sufficiently large to support the original structure of impulsivity and included more males than females. Second, we only examined the internal reliability of the scale; test–retest reliability was not investigated. Third, we only explored the relationship between impulsivity trait and choice; associations among impulsivity traits, choices, and actions should be explored to better understand the nature of impulsivity. Fourth, we only compared the impulsivity between smoking and nonsmoking and between drinking and nondrinking; the association between impulsivity and the problematic use of cigarettes and alcohol was not estimated. Therefore, we highlight the need to clarify the association between impulsivity and substance use disorders.

## Conclusions

The study supported the reliability of the scores of the UPPS-P impulsive behavior scale; however, the structure validity was not acceptable in our sample. Regarding validity, positive urgency was negatively related to the Beads task, and negative urgency and positive urgency were positively related to the DDT and BART. Moreover, impulsivity was strongly related to depression and anxiety; sensation seeking was higher in males than females and in drinkers than nondrinkers; and lack of premeditation and lack of perseverance were higher in nonsmokers than smokers. Further studies are needed using large samples to verify the structure validity of the UPPS-P scale.

## Data Availability Statement

The raw data supporting the conclusions of this manuscript will be made available by the authors, upon reasonable request, to any qualified researcher.

## Ethics Statement

The studies involving human participants were reviewed and approved by all procedures followed were in accordance with the ethical standards of the responsible committee on human experimentation (institutional and national) and with the Helsinki Declaration of 1975, as revised in 2008. Informed consent was obtained from all participants. The patients/participants provided their written informed consent to participate in this study.

## Author Contributions

JD revised the Chinese version of the UPPS-P, ZZ, HZ, and CZ recruited participants, and QR and XQ tested participants and collected the data. XQ completed the scales data entry. VV provided the data of impulsivity choice tasks. CZ and WL designed the study. YZ analyzed the data of scales and wrote the manuscript with the inputs from all authors.

## Funding

This study was supported by the Shanghai Science and Technology Commission International Cooperation Project(STCSM, grant number 18410710400), Cooperative Research Project of Translational Medicine Collaborative Innovation Center (grant number TM201801), 2018 Shanghai Municipal Education Commission–Gaoyuan Nursing Grant Support (grant number Hlgy1804kyx) and the 2017 Shanghai Jiao Tong University School of Medicine Doctoral Innovation Fund (grant number BXJ201705).

## Conflict of Interest

The authors declare that the research was conducted in the absence of any commercial or financial relationships that could be construed as a potential conflict of interest.
